# Barriers and facilitators to healthcare access for children with disabilities in low and middle income sub-Saharan African countries: a scoping review

**DOI:** 10.1186/s12913-019-4822-6

**Published:** 2020-01-06

**Authors:** Molalign B. Adugna, Fatima Nabbouh, Selvia Shehata, Setareh Ghahari

**Affiliations:** School of Rehabilitation Therapy, Queen’s University, Kingston, Canada

**Keywords:** Sub-Saharan Africa, Barriers, Children with disabilities, Health services, Healthcare access

## Abstract

**Background:**

Children with disabilities (CwDs) make up around 150 million of the billion people with disabilities in the world. The Sub-Saharan African countries have a large number of CwDs who have limited access to healthcare and rehabilitation care. This, combined with chronic poverty, low education, and inadequately trained healthcare professionals, substantially lowers these children’s quality of life. The main objective of this scoping review was to discover the barriers and facilitators to healthcare access for CwDs in selected low to middle income Sub-Saharan African countries. As African countries significantly vary in socioeconomic status, we only focused on countries in Sub-Saharan Africa who allocated less than $50/person to healthcare.

**Methods:**

A broad and iterative search strategy using multiple sources and databases including CINAHL, Medline, Global Health, and Embase were utilized. Using a comprehensive search strategy, 704 articles were generated. After removal of the duplicates, 466 of them were screened based on the study inclusion and exclusion criteria. After iterative reading and screening of these articles, a final 15 articles were included in this review.

**Results:**

This scoping review shows that CwDs in the selected Sub-Saharan African countries face major barriers including stigma and negative attitudes, poverty and insufficient resources, inadequate policy implementations, physical inaccessibility, lack of transportation, lack of privacy, and inadequately trained healthcare professionals to deal with disability. Emotional and social support, including peer support for CwDs and caregivers, were identified as facilitators for better access to health services.

**Conclusions:**

There is limited access to healthcare services in the low and middle income Sub-Saharan African countries due to poverty, low education, inadequate healthcare systems, and shortage of healthcare professionals. It is evident that there are socioeconomic, cultural, and physical related impediments that affect CwDs’ and their caregivers’ access to the required healthcare services. Policy development, improved physical accessibility, public disability awareness, and parental support are some of the key facilitators to access healthcare services. The study highlights the importance of revisions to childhood disability and healthcare provisions policy and practice as well as sustainable rehabilitation programs. Further research is required to explore ways to improve experience of accessing health services.

## Background

The United Nations International Children’s Emergency Fund (UNICEF) reported that the global rate of child disability is high and there are approximately 150 million children with disabilities (CwDs) [1, 2]. The Sub-Saharan Africa is home to a large number of CwDs, many of whom lack access to basic healthcare services [3].

International organizations such as WHO and UNICEF work to address the needs of children in underdeveloped countries and war-torn countries [2, 4]. WHO declares [1] the support for people with disabilities as a human rights issue [2] and describes mainstreaming disability as an important development issue. However, there is a lack of disability awareness, services, and research in almost all of the Sub-Saharan African countries [4] . UNICEF expresses that CwDs often do not survive their childhood or for those who do survive, their conditions become worse due to lack of basic primary healthcare service provisions.

Many CwDs and their families do not have equal access to healthcare services compared to children without disabilities, do not undergo treatment or disability-related services, and are overall excluded from everyday life activities [4] . Basic healthcare services are defined as the least healthcare needed to maintain sufficient health and protection from disease [5] . Access to healthcare services exists when services are available in enough supply and when there is an opportunity to receive healthcare. Barriers to health services can arise when there are financial, organizational, social, or cultural strains within the community [6]. In many Sub-Saharan African countries, accessing healthcare services and rehabilitation services for CwDs is limited to urban areas, if available at all [3]. When children miss out on essential treatment for basic illnesses such as fever and diarrhea or miss vaccinations, these treatable illnesses often evolve into lifelong disabilities [2]. Childhood disability in the Sub-Saharan Africa is closely linked to poverty [3]. When impoverished children experience disability, it is often due to inadequate healthcare services for pregnant mothers and basic healthcare services for young children [5]. Disability persists and is continuously affected by socioeconomic, political, and cultural situations of a given society [3].

There are currently no scoping reviews on the barriers and facilitators affecting healthcare access for CwDs in the low and middle Sub-Saharan African countries. Therefore, it is important to explore and analyze the existing literature and compile the information in one source for easy access and to develop the next steps for intervention.

## Methods

The outline for this review followed the five-stage framework described by Arksey and O’Malley [7]: 1) identifying the research question, 2) identifying relevant studies, 3) study selection, 4) charting the data, and 5) accumulating, summarizing and reporting the results. This paper aimed to answer the following research question: “What are the facilitators and barriers to healthcare access for CwDs in selected low and middle Sub-Saharan African countries?”. For the purpose of this paper, access to health care was defined as receiving appropriate health care resources for the purpose of maintaining or improving health status [6]. The term “children with disabilities” refers to children up to the age of 18 who have “long-term physical, mental, intellectual, or sensory impairments which in interaction with various barriers may hinder their full and effective participation in society on an equal basis with others” [8, 9].

### Data sources and search

Multiple electronic databases including CINAHL, Medline, Global Health, and Embase were used to search for relevant studies between March–July 2017. Manual search was completed using Google Scholar and Queen’s University Library search engines. Search terms included: “children with disabilities”, “barriers to healthcare access”, “African children healthcare access”, “facilitators to healthcare access”, and “barriers and facilitators to healthcare access”. The selected articles were combined into EndNote Basic and duplications were deleted.

### Study selection and screening

The search resulted in 704 records. Articles were screened based on their title and abstracts for eligibility and imported into EndNote Basic to remove duplicates. Through independently screening by the reviewers, the remaining studies were then examined further by reviewing their abstracts and removing articles that did not satisfy the inclusion criteria. The remaining papers were then screened for a final time through a full-text revision and non-relevant papers were excluded. There was no restriction on publication date due to limited research on the topic. Finally 15 peer-reviewed articles were included in this scoping review.

Articles were included if they discussed barriers or facilitators to healthcare access for children under the age of 18 in selected low and middle Sub-Saharan African countries. As the African countries significantly vary in terms of their socioeconomic status, this review only focused on countries in Sub-Saharan Africa who allocated less than $50 to healthcare per person. This reference point was used to focus on the countries that were most in need of health resources. This provides useful information about whether the government spends enough of its own resources on health in order to provide universal coverage of essential healthcare services, particularly for vulnerable groups such as children.

### Data charting and abstraction

A PRISMA chart and a standardized data extraction form were used. A two-step charting process was completed using Excel. The first step included creating a table and extracting the following information from reading the abstracts of the identified articles: authors/researchers of the article, publication year, study objectives, whether the article was about Africa, included children 0–18 years of age, included children with disability, and collected data on access to healthcare service. The next step of the charting process included extracting the following information from the remaining papers by reading the full articles: authors/researchers of the article, publication date, objectives of the paper, inclusion criteria, age and gender of the children, sample size, age, income and education of parents, gender of the children, children’s education, type of disability, city, country, and setting of the study, study method, outcome of the paper, results, facilitators and barriers to healthcare services.

### Data analysis

The next step of the process included an iterative independent article screening, reading, and theme coding. Constant discussions on the procedures and emerging issues took place until the researchers agreed and major themes were formed. A card system method [10] was used to come up with overarching themes and categories across all reviewed articles and synthesized results from reviewed studies. Finally, the major themes that emerged from the data regarding facilitators and barriers to healthcare access to CwDs in Sub-Saharan African countries were identified and discussed.

## Results

### Study screening results

**Fig. 1:**
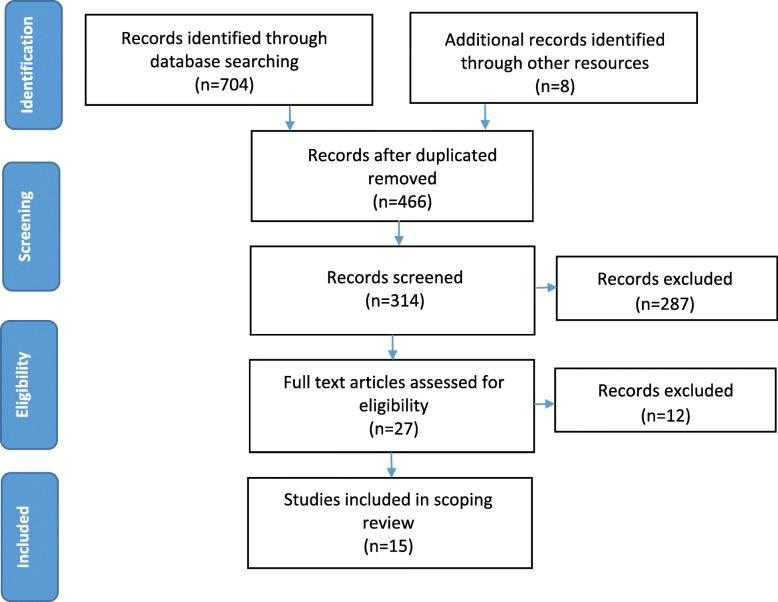
PRISMA flow diagram for the scoping review process

A total of 466 articles were imported to EndNote, including articles that were identified through manual searches. After excluding articles by examining the title, 314 article abstracts were screened and 27 articles remained for full-text examination. After final evaluation, 12 more articles were excluded. Nine qualitative studies, five mixed design studies, and one quantitative study met all inclusion criteria and were used in the scoping review for a total of 15 articles. See Fig. 1 for full article screening process.

Although there were 29 countries in Africa that allocated less than $50 per person to healthcare, papers included in this study were from only nine Sub-Saharan countries. Of the 15 articles discussed in this review, four papers reported on Ethiopia [11–13], two on Zimbabwe [14, 15], two on Malawi [16, 17], two on Cote d’Ivoire [18, 19], one on Rwanda and Uganda [20], one on Uganda [21], one on Kenya [22], one on Zambia [23] and one on Nigeria [24] (Table 1 in Appendix).

After extraction of data and comparing and contrasting the results, several themes emerged regarding barriers and facilitators to health care access for CwDs in low and middle Sub-Saharan African countries (see Additional file 1 for more details).

### Trends in quantitative and qualitative studies

All 15 articles identified barriers to accessing healthcare services for CwDs in selected Sub-Saharan African countries. Fourteen studies utilized qualitative methods, including mixed methods, and relied on interviews or questionnaires to identify specific barriers. Thirteen of those 14 articles determined these barriers through interviews and group sessions with the CwDs’ caregivers (parents, or any other person who was the main person to take care of the child) or service providers and only one study interviewed the children themselves [20].

### Theme 1: Attitudes, beliefs and awareness of disability

#### Stigma

Stigma, including experienced stigma and fear of stigmatization, was a reported barrier in five papers. High levels of stigma were reported by many families of children with disabilities, preventing them from allowing their children to access health care and proper medical treatment [11, 12, 15, 23] . Caregivers in Ethiopia, Zimbabwe, and Zambia expressed concerns about being treated poorly by others and how this caused them to decide to hide their child’s condition instead of seeking treatment [11, 12, 15, 23]. A health extension worker in Ethiopia noted that caregivers were often worried about being treated differently, feeling ashamed or embarrassed about their child’s condition, and made efforts to keep their child’s condition a secret [11]. Families were worried that if others knew that their child had a disability, they might never get married due to stigma [12].

#### Personal and community attitudes

Negative attitudes towards CwDs can affect access to healthcare services and facilities. Negative attitudes both from caregivers and from healthcare professionals hinder accessing healthcare services [11, 17, 20, 21] . For example, a study showed that negative attitudes about disability in Ethiopians resulted in lack of support from the community and the children’s families’ which decreased the motivation of health extension workers [11]. Another example of the negative impact of attitude on health access is seen in Malawi where caregivers believed “nothing can be done” for their child and therefore did not seek medical treatment [17].

Personal attitudes and beliefs of the healthcare staff in Uganda and Rwanda affected the youths’ access to healthcare services [20]. One participant in Uganda reported a case where a nurse would not provide HIV related services such as blood tests to a girl with a physical disability due to misconceptions that youth with physical disabilities were not sexually active [20]. Furthermore, caregivers reported that they were sometimes rejected by healthcare workers and their children did not receive treatment because some healthcare providers believed that these children could not be treated [13, 15, 23] . A study by Bannick and Stroeken [21] found that parents of CwDs were often discouraged from seeking healthcare services for their child as it was perceived as a waste of money. Parents in this study were advised by others in the community to leave their child to die or that their child was “already dead [21].

Despite the fact that there were challenges and barriers that prevented accessing healthcare by CwDs, a positive attitude among family and community members can encourage caregivers to seek treatment for their children. Family members were more likely to encourage parents to seek treatment for their children when they noticed an improvement in the child’s health as this gave them a sense of hope. Bannink et al. (2015) and Nota et al. (2015) found that when a child showed visible and observable health improvements, such as surgery recovery or significant rehabilitation progress, the community’s attitudes changed and became more positive and supportive [15, 21].

To counteract the attitude of caregivers, the literature suggested making treatment sessions more attractive to the caregivers. Having longer therapy sessions was reported to be a facilitator for attending therapy sessions in Zimbabwe [15]. Nota et al. (2015) found that caregivers were less likely to miss their child’s therapy sessions if they were group sessions as opposed to individual sessions. The authors suggested that the group setting allowed caregivers to share their experiences with each other and with the healthcare workers as well as compare their child’s progress to other children. These interactions would also create meaningful relationships between the caregivers and the healthcare workers, allowing for better treatment outcomes for the children and therefore encouraging parents to continue with the treatment [15].

#### Lack of awareness

Disability awareness refers to factors that associate with being educated on and understanding disability. Attitudes were considered distinct from awareness in that awareness is related to education while attitudes were related to personal or cultural beliefs. A well-informed community about disability and how to identify children with disabilities (i.e. a higher awareness), would provide an encouraging context for children accessing services.

The literature showed that a lack of disability awareness acted as a substantial barrier in the community to seeking treatment. Many caregivers in Ethiopia lacked awareness of their CwDs. They did not recognize their children as people with disabilities or they did not acknowledge their children’s needs of seeking medical attention [12, 13]. Some caregivers believed their child was disabled due to head injury, birth complications, epilepsy, or religious causes. As a result, a large number of caregivers sought out traditional places for care rather than medical attention [13]. Shibre et al. (2001) found that many caregivers only relied on prayer to address problems of their child’s disability [12].

Lack of awareness affected health care professionals as well. For example, health extension workers in Ethiopia expressed the need for in-service training to identify causes, prevention, and treatment for children experiencing mental health and developmental disorders [11]. They described having poor knowledge on child developmental disorders and reported that this lack of skills and knowledge lowered their confidence when working with the children and their families [11].

According to Tekola et al. (2016), awareness-raising activities of two autism centers in Ethiopia, the Joy and the Nehemiah, helped to educate and inform the public and the healthcare staff about the illness, conducting diagnoses and decreasing stigma, thereby increasing access to healthcare services [25]. However, the centers were limited to children with autism spectrum disorder and located in the capital city of Ethiopia [13]. Bannink et al. (2015) also stressed the importance of making the community more aware and informed about childhood disability. Being more informed about causes and etiology of the disease or disability helped to change the community’s beliefs and perceptions and gain support for children with disabilities and thereby reducing stigma [21]. There was evidence that educating caregivers about the child’s condition can also positively impact access to health services. CwDs were often neglected by caregivers and not taken to healthcare facilities and rehabilitation appointments [17]. Paget et al. (2015) found that caregivers were less likely to neglect their children when they were educated about their condition [17]. This awareness allowed parents to be more informed, understanding, and accepting, which increased their likelihood of seeking and continuing treatment for their children [17, 21]. However, it is important to note that visible health improvements in children were a stronger facilitator, compared to awareness, in changing attitudes. Bannink et al. (2015) reported that even when family members were informed about the child’s disability and rehabilitation treatment, their attitudes did not change until they saw noticeable improvements in the child’s health condition [21].

#### Cultural beliefs

Cultural beliefs also prevented CwDs from accessing health care services [13, 19, 21, 23]. Studies reported on the belief among African communities that having a child with a disability was a punishment from God or the child was possessed by the devil [13, 19]. In Ethiopia and Zambia some people believed that disability was a source of witchcraft, curses, and bad luck [13, 19, 21, 23]. These beliefs prevented parents and caregivers from seeking health care treatment for their child. In Uganda, mothers of children with spina bifida and hydrocephalus were feared in their communities as they were seen as having given birth to a demon [21]. The mothers were discouraged from taking their child to the hospital and from engaging in vital practices such as breastfeeding. Instead, parents were encouraged to take their children to witchdoctors to remove the curse [21]. Bannick et al. (2015) also described a common practice in Northern Uganda where mothers would carry their infants on their backs, go to the river, and untie the wrap carrying the child. The death of the child would then be reported as an accidental drowning [21]. Similarly, in Cote d’Ivoire, CwDs were often isolated and in some cases killed out of fear of harming others [19]. Paget et al. (2016) found that CwDs were at risk of parental abuse and neglect [17]. Healthcare workers in the study indicated that neglected children were often left alone and locked up, not cared for, not fed, and not taken to healthcare services [17]. Some healthcare professionals in Cote d’Ivoire and Uganda were reluctant about the idea of providing CwDs with healthcare services due to the cultural beliefs surrounding the etiology of disabilities [19, 21]. In Uganda, some healthcare workers refused to treat children with spina bifida and hydrocephalus as they preferred not to deal with what they referred to as “special cases” [16, 21].

### Theme 2: Accessibility of services and systems

#### Availability of health services, clinical pathways, and qualified healthcare staff

Available research showed that CwDs in Zambia, Malawi, and Kenya have limited access to healthcare services including community and hospital services in populated areas [16, 22, 23]. Therefore, caregivers would take their children to be treated by traditional healers instead of seeking medical attention [23].

Lack of qualified healthcare professionals had a significant impact on the quality of the healthcare services, which can deter caregivers from using those services. In Ethiopia, for example, a large number of children with autism and intellectual disability remained without official and validated diagnoses due to lack of awareness, education, and training for healthcare professionals and staff [13]. Tekola et al. (2016) and Tilahun et al. (2016) indicated that there was inadequate medical treatment for children with autism and intellectual disabilities in Ethiopia where the population is over 100 million, with only two treatment centers located in the capital city, Addis Ababa [13] . In 2016, there were only two child psychiatrists in Ethiopia and no psychologists were trained in child mental health [13]. Yousafzai et al. (2005) found that communication was a barrier for deaf teens in Uganda when trying to access HIV healthcare service [20]because most healthcare workers did not know sign language. This lack of training for the staff prevented teens with disabilities from being able to communicate with healthcare staff and therefore from accessing services [20]. In Malawi and Uganda, similar findings were observed in which healthcare workers lacked awareness of services and did not have access to standardized assessment procedures for child mental health [17, 21].

Healthcare workers were often unclear about referral pathways and displayed poor communication skills. Therefore, when healthcare was needed, it was not adequately provided to these children [17, 20, 21]. It was unclear within the articles what these services and standardized assessment procedures should be. Furthermore, in Malawi, caregivers expressed their concern about not finding services for their children’s specific disability, such as an ear clinic for hearing disabilities and physiotherapy for physical related disabilities. They also described not having health facilities in the area they lived in, thereby making it difficult to even find a service [16]. In Kenya, although physiotherapy was present within health-based rehabilitative services, adults with disabilities were prioritized and inadequate service was provided to children experiencing disability [22]. In Zambia, due to lack of related policies and processes, it was not possible to properly plan to identify, administer care and follow up with children experiencing disability. Health care workers also blamed heavy workloads, limited staffing, and inadequate training for inadequate care [23]. Other researchers found that there were very little rehabilitative staff and lack of health care workers, making it extremely challenging to provide access to healthcare services to all CwDs [11, 17, 22, 23]. In Zimbabwe, Nota et al. (2015) also found that the availability of the rehabilitation staff was a facilitator for caregivers to access the rehabilitation services. Caregivers were more motivated to access healthcare services if the rehabilitation workers were available to provide required services every time the children visited [15].

The literature suggests three ways to change the process of care to improve health access. First, allowing caregivers and CwDs to be active participants in the process of care as opposed to passive recipients. When parents and children had a say in the treatment process, they were more likely to be motivated to access those services [15, 21]. The second way to improve health access is by using an interdisciplinary approach throughout the process of care. When professionals from different disciplines collaborated, they were more likely to provide the best possible care for the child and this in turn motivates caregivers to bring their children to those healthcare services [21]. Finally, using a holistic approach in which all rehabilitation services are offered in one location, such as in the same hospital, can help improve health access. Caregivers in Zimbabwe noted that this was a motivator for them to access those services [15].

Magnussen and Ingstad (2011) found that one of the reasons caregivers of CwDs did not access [15] healthcare services in Zambia was due to a poor reputation of the healthcare facility [23]. As a result in some cases, although the facility existed, it might not be used by families of CwD because of poor reputation. In tightly knit communities, the opinions of the community impacted the caregivers’ opinions and how much they used those facilities. The study reported that this barrier was overcome through the use of neighborhood health committees and community volunteers in Zambia who helped identifying CwDs in the community. According to Magnussen and Ingstad (2011), access to healthcare facilities in Zambia was improved by enhancing the reputation of those health centers. Positive experiences shared with others changes the community’s attitudes about the facility and motivates others to access those services [23].

#### Availability of resources, equipment, and funds

Research showed that limited funding and resources negatively impacted care for CwDs. Many healthcare facilities were underfunded and this in turn affected the access by CwDs [11, 16, 18, 22]. For example, in Kenya, staff described that as a result of limited funding, CwDs had limited access to effective services because of low staffing, low quality resources, and old equipment [22]. In Malawi, Paget et al. (2006) identified that healthcare workers tried their best to provide treatment and service to CwDs and their caregivers, however, they faced lack of space to provide treatment, lack of equipment to assess and transport children, and limited access to medicine to provide to children who were medically unstable [17]. Devendra et al. (2013) reported that in Malawi, only 5% of 98 CwDs who attended rehabilitative services received rehabilitative equipment. Having enough funding was a facilitator for better access to services. Most caregivers believed that if their children had access to equipment such as hearing aids, wheelchairs, bicycles, crutches, walking aids, and glasses, it would help with their treatment and improve their access to service [16].

### Theme 3: Physical environment

#### Transportation and weather conditions

Travelling with a child with a disability can be quite difficult for caregivers, especially if the child used heavy assistive devices such as a wheelchair or if the family lived in rural areas far from the health facility. This can hinder families from accessing those services or from taking their child with them to medical appointments [14, 15, 23].

Parents expressed difficulty travelling with their disabled children as they would have to carry them on their backs [14, 15, 23]. In Zambia, healthcare services were scattered in rural areas and therefore people had to walk long distances to access services [23]. It was especially difficult to get around during and after poor weather conditions such as heavy rainfall. Roads were often blocked and became inaccessible due to the heavy rainfalls and floods. Therefore, this prevented people from accessing and using healthcare services in those areas [23]. Health facilities in Malawi [16, 17], Ethiopia [13], Uganda and Rwanda [20], and Kenya [22] were also far apart and caregivers of CwDs, especially those living in rural areas, struggled to access them due to travel distance, difficulty travelling with their children, or high transportation fees. In Uganda and Rwanda, many health facilities were far away and youth with physical disabilities struggled to access them as they couldn’t walk the distance [20]. In Ethiopia, a child can only receive a formal autism diagnosis from government or private mental health clinics in Addis Ababa. However, the government clinics could only be accessed after a referral and families living in remote areas often did not have access to a referral process. Those who managed to access referrals were often unable to travel the long distance to these clinics [13]. Finkenflugel and Van Maanen (1996) found that this barrier was overcome in Zimbabwe through the use of community-based rehabilitation (CBR). Since CBR took place in the child’s home, the service was provided more effectively than using traditional healthcare facilities [14].

#### Physical inaccessibility

Physical barriers also can hinder CwDs accessing healthcare. Yousafzai et al. (2005) found that teens with physical disabilities in Uganda were not able to attend community healthcare meetings regarding HIV because the locations were physically difficult to access. In Rwanda, it was reported that children with physical disabilities were unable to visit health facilities because they were impossible to access [20]. These authors, however, did not report what made the environment physically inaccessible.

Physical inaccessibility was not only about getting in and about a facility, rather, was also about using the available resources. Research showed that resources such as information leaflets, flyers, and print materials was not accessible by CwDs and this limited their access to services as they were not able to attain the information being communicated [20]. For example, information about healthcare services conveyed over the radio was not accessible by deaf teens in Uganda and Rwanda. Similarly, posters were not accessible by children with visual impairments in Uganda and Rwanda. These communication barriers prevent CwDs from knowing about and therefore accessing those healthcare services [20]. Providing information in alternative format was suggested as one of the solutions.

### Theme 4: Social factors

#### Poverty

Poverty was reported to be a recurring barrier for CwDs and their families. Caring for a CwDs can be expensive as they may require special food, assistive devices, and transportation fees for medical appointments [15]. Tilahun and Hanlon surveyed caregivers in Ethiopia and found that one of the most unmet needs was lack of financial support. Without financial support from family, the government, or other organizations, it was difficult for CwDs to access healthcare services [13]. Limited funds can prevent caregivers from paying for private services and transportation fees [15, 16, 20, 22, 23], healthcare fees/bills [21, 22], assistive devices [21] and more. Families living in poverty had to ration and prioritize their spending and they often could not afford expenses to healthcare of their CwDs [17, 23]. Health extension workers in Ethiopia indicated that families experienced challenges such as transportation and treatment costs that limited their access to proper treatment [11]. Nota et al. (2015) found that caregivers in Zimbabwe experience financial difficulties that prevented them from accessing healthcare and made them more likely to miss their child’s scheduled therapy appointments [15].

Attending health related visits can threaten the family breadwinner’s employment. In Zimbabwe and Zambia, caregivers found it difficult to access healthcare services because it was difficult to ask for time off of work, especially to attend several therapy sessions [15, 23]. They further described that there were long wait times at the children’s rehabilitation clinics that impacted the parents’ job [15]. This was especially difficult for families experiencing poverty and single caregivers [23].

Lack of financial support from the family was another financial aspect to consider. Caregivers described that fathers would often abandon the family of the disabled child and would not support the child financially [21]. This made it difficult for the child to receive medical attention as it caused financial and social strain on the mother, who would become the sole provider for the child.

Financial support from the government or organizations was a strong facilitator to accessing healthcare services, especially for economically disadvantaged families [15]. A study conducted in Zimbabwe found that caregivers of children with congenital diseases were more motivated to take their children to therapy sessions if incentives, such as toys, transportation money, food, assistive devices, and clothes, were given at the rehabilitation department [15]. These incentives allowed the child to be more functionally independent and decreased the financial burden on the caregiver.

#### Lack of privacy

In Uganda and Rwanda, lack of privacy was a major concern for adolescents with disability to access health care especially for those with hearing impairments [20]. Adolescents explained that they often needed a helper to assist them to medical appointments and this breach of privacy often prevented them from going to their appointments. They also described that although confidentiality was acknowledged by the healthcare staff, they were concerned that the third person (i.e. the assistant) would gossip [20].

#### Peer support

Parents and caregivers were more likely to access healthcare services and continue treatment for their children if they received emotional and psychosocial support from friends and family members [13, 15, 23]. In Zimbabwe, caregivers reported being motivated to attend therapy sessions by receiving emotional support from other caregivers of CwDs [15]. Research also showed support of peers was important to both CwDs and their caregivers. Children had better access to health care services when other family members provided social support [23]. In addition, peer support was a common coping strategy for caregivers of CwDs [13].

The use of support groups for caregivers was a facilitator for accessing healthcare services [17, 21]. In Malawi, health care workers noted that support groups helped inform and empower caregivers of CwDs. The groups provided parents with emotional and psychosocial support that helped them cope with their child’s illness [17]. The health care workers also suggested that inviting family members to these peer support groups may help to raise awareness and decrease stigma [17]. Similarly in Uganda, parent support groups allowed parents to encourage and empower each other to fight the stigma in their communities [21]. Parents also noted that the support they received from the parent support groups helped them feel less worried about their child’s condition [21].

## Discussion

The present review identified 15 peer-reviewed articles that discussed healthcare access for CwDs in Sub-Saharan African countries that allocated less than $50 per person on healthcare spending. This review is the first scoping review to examine barriers and facilitators to healthcare access for CwDs in Africa. Having identified these factors, recommendations can then be put forth for developing intervention plans.

The findings of this review suggest that there are commonly reoccurring factors preventing caregivers from accessing healthcare services for their children in Sub-Saharan African countries. These factors were identified in different communities across the selected countries where the reviewed studies were conducted. Facilitating factors to healthcare access were not as evident in the literature as barriers, which may be due to limited scientific research and publications on this topic and the lack of intervention in these countries.

Poverty was the most reported barrier preventing CwDs and their caregivers from accessing healthcare services; it was discussed in 6 papers in 5 different countries. In Nigeria, for example, poverty was the most influential factor affecting healthcare service use for children and their mothers [26] . Most healthcare services were not free, even in missionary hospitals where it was free in the past [27]. The conditions of the hospitals were poor as they were usually unclean and crowded and lacked funding and medical staff, with much of the work being carried out by overworked nurses. When families can barely afford to live, taking their CwDs to therapy may not be a priority for them. Shillingford (2010) reported that people in Uganda could not even afford to buy food [27]. In Uganda, individuals were not admitted to the hospital unless they were accompanied by a relative who will regularly take care of them by feeding and bathing them during their stay at the hospital. Therefore, individuals who were unable to afford to take care of themselves may be reluctant to take their family members to the hospital. Nota and Chikwaha suggested that providing incentives such as toys, transportation money, and disability aids may encourage caregivers to bring their CwDs to health facilities [15].

It is evident that religious and supernatural beliefs regarding the etiology and treatment of disabilities can affect service access and delivery. This was perhaps one of the most powerful barriers to healthcare access in Africa. Most papers in this study identified cultural beliefs regarding the causes of disability, such as witchcraft, religious punishment, and possession of evil forces [11, 13, 17, 19–24]. These beliefs were reported in 8 different countries. Although not all papers identified a direct link between these beliefs and accessing healthcare services, there was a clear association between the two. If a family believed their child’s condition was of supernatural cause, they may never seek medical treatment, no matter how adequate the healthcare staff were. Many families holding these cultural beliefs looked for traditional rather than medical treatment, such as through prayer or cultural rituals. Aldersey (2014) noted that families in Kinshasa, Congo who viewed the causation of intellectual and developmental disabilities as biomedical were more likely to seek medical treatment for their children [28]. Families who believed in supernatural causes of their child’s disability often sought out traditional healers or did not seek treatment at all. These beliefs also heavily contributed to stigma and this therefore could prevent families from being open about their children’s conditions and from seeking treatment. In the literature, stigma was repeatedly found to force families to hide their children, conceal their children’s condition, and prevent them from leaving their homes to access a healthcare service or needed treatment. Aldersey (2014) found that families of children with developmental disabilities in Kinshasa hid their children at home and out of sight in order to prevent others from thinking the disability was a punishment to the family for participating in supernatural practices [28].

In addition to the discussed barriers, other factors can also affect access to healthcare services. A study on healthcare access for typically developing Nigerian children found that family size had a direct effect on children’s attendance to a health clinic, with higher attendance for smaller families [29]. The age of the mother also affected attendance. Younger mothers were more likely to take their children to a clinic for care than older mothers. More educated parents were also more likely to take their children to clinics for immunization than illiterate parents. Perception of illness severity also affected whether or not mothers took their children to health clinics. Mothers were more likely to take their children to health clinics for fevers than for skin conditions. Although these findings were for typically developing children in Nigeria, these factors may also affect CwDs and therefore further studies are needed.

Many papers also highlighted the importance of coping strategies for caregivers and families of children with disabilities. Many caregivers in Malawi and Ethiopia drew on sociocultural and spiritual coping strategies to help them obtain hope and meaning [13, 17]. Rehabilitation staff can have a significant role in supporting families to cope with needs of care of a child with disability by being mindful of and understanding their patients’ social and cultural environment and incorporate this into the goal-setting process [30].

### Strengths and limitations

A comprehensive literature search was conducted from multiple sources and databases including grey literature for the inclusion of all types of studies from low and middle Sub-Saharan African countries. However, there were some limitations in this research study. There was limited access to some articles because they were not available online. Similarly, some authors did not publish their research or only published the abstract, which minimized the overall content of the scoping review. Research on healthcare access for CwDs in Sub-Saharan Africa is already scarce. Only articles published in English were included. Thus, this may have limited findings of the study. Due to the limited research, articles were not excluded by publication date and therefore the articles in this review ranged from 1996 to 2017. For this reason, another limitation might be outdated information. However, the themes found in the older articles were also seen in more recent papers.

### Implications for childhood disability policy and practice

It is evident that there is a lack of scientific information about CwDs and their access to healthcare services in the Sub-Saharan African countries. This may be due to the lack of childhood disability research and interventions. There is also low attention about CwDs from the government that affects the provisions of healthcare services for CwDs. The World Health Organization (WHO) also recommended developing ways to address funding barriers for rehabilitation and healthcare services [4]. As this study shows differences in barriers and facilitators, it can indicate future steps or recommendations to inform disability awareness and policy along with intervention strategies on childhood disability and healthcare provisions. Moreover, the study findings would allow development of sustainable programs such as CBR interventions, which are needed in low and middle Sub-Saharan Africa countries, to remove barriers and increase facilitators to healthcare access for CwDs.

## Conclusion

Attitudinal problems, poverty, inadequately trained healthcare professionals, and physical inaccessibility are frequently reported as major barriers to healthcare access for CwDs in the low and middle income Sub-Saharan African counties. Whereas, efforts for policy development and improving physical accessibility, public disability awareness, and professional and parental support are key facilitators. Healthcare professionals working with CwDs should be aware of these barriers and facilitators in order to develop effective assessments and intervention plans. To include these children in the healthcare process, there needs to be a greater presence of healthcare professionals, resources, and modifications to healthcare systems. Further research on pediatric health and childhood disability is needed for enhancing evidence-based practice in the Sub-Saharan Africa. More research is needed to further examine access to healthcare for CwDs in the low and middle Sub-Saharan Africa from the broad perspective of stakeholders including children themselves, parents or caregivers, and healthcare professionals. Thus, future research should investigate recommendations for enhancing access to healthcare services for CwDs in these countries so that action plans and interventions can be designed and put in place.

### Supplementary information

**Additional file 1.** A: Reviewed Articles by Selected Country. B: Major Themes of the Findings. C: Barriers to Healthcare Services Access for CwDs. D: Facilitators to Healthcare Services Access for CwDs

## Data Availability

Not applicable.

## Appendix

**Table 1:** A summary of characteristics of the reviewed articles and their reference to the selected countries

Study	Year	Study Design	n	Diagnosis	Country	Barriers Present	Facilitators Present
Tekola et al. [25]	2016	Qualitative	10	Autism	Ethiopia	√	√
Tilahun et al. [13]	2016	Qualitative	102	Autism, Intellectual disability	Ethiopia	√	√
Shibre et al. [12]	2001	Quantitative	178	Schizophrenia Affective disorder	Ethiopia	√	
Miftah et al. [11]	2017	Mixed method	104	Mental Health	Ethiopia	√	√
Finkenflugel et al. [14]	1996	Qualitative	75	Neurological: Cerebral palsy	Zimbabwe	√	√
Nota et al. [15]	2015	Qualitative	40	Neurological and Orthopedic	Zimbabwe	√	√
Paget et al. [17]	2016	Qualitative	24	Neurodisability	Malawi	√	√
Devendra et al. [16]	2013	Mixed method	296	Vision, Hearing, Physical, Learning, Seizure	Malawi	√	
Bayat [19]	2014	Qualitative	65	Developmental	Cote d’Ivoire	√	
Alloh et al. [18]	2009	Mixed	8	Psychomotor	Cote d’Ivoire	√	√
Yousafzai et al.	2005	Qualitative	123	Deaf, Blind, Physical Communication	Uganda and Rwanda	√	
Bannink et al. [21]	2015	Qualitative	178	Spina Bifida Hydrocephalus	Uganda	√	√
Bunning et al. [22]	2014	Mixed method	N/A	Varied	Kenya	√	√
Magnussen et al. [23]	2011	Qualitative	29	Epilepsy, Cerebral Palsy Hydrocephalus, Congenital limb, Seeing, Hearing, Developmental, Down syndrome	Zambia	√	√
Ogunkeyede et al. [24]	2017	Mixed method	155	Hearing impairment	Nigeria	√	

## Abbreviations

CwDs: Children with Disabilities
UNICEF: The United Nations International Children’s Emergency Fund
WHO: World Health Organization
