# Supplementary material for: Barriers and facilitators to healthcare access for children with disabilities in low and middle income sub-Saharan African countries: a scoping review
Source: BMC Health Serv Res. 2020 Jan 6;20:15. doi: 10.1186/s12913-019-4822-6 (PMC6945633; doi:10.1186/s12913-019-4822-6)
Supplement: Supplementary file 1 — Additional file 1. A: Reviewed Articles by Selected Country. B: Major Themes of the Findings. C: Barriers to Healthcare Services Access for CwDs. D: Facilitators to Healthcare Services Access for CwDs [file 12913_2019_4822_MOESM1_ESM.docx]

Additional file 1: **A: Reviewed Articles by Selected Country**

| **Country** | **Reference** |
| --- | --- |
| Cote d’Ivoire | (18, 19) |
| Ethiopia | (11-13, 25) |
| Kenya | (22) |
| Malawi | (16, 17) |
| Nigeria | (24) |
| Uganda | (20*-21) |
| Rwanda | (20) |
| Zambia | (23) |
| Zimbabwe | (14-15) |

***Note: (20) discussed conditions in Uganda and Rwanda**

**B: Major Themes of the Findings**

| **Category** | **Category Definition** | **Barriers** | **Facilitators** |
| --- | --- | --- | --- |
| Personal attitudes and beliefs | Attitudinal factors, such as stigma and cultural beliefs, can affect access to health services especially in tight-knit communities | Stigma  Cultural beliefs  Negative attitudes | Hopefulness regarding treatment |
| Services and systems | Factors that affect access to healthcare due to aspects of the healthcare system, health services, or government policies | Lack of health services  Lack of qualified professionals  Inadequate health care professionals  Lack of resources and equipment  Bad reputation of health facility | Community workers and volunteers  Caregivers and children are active participants in the care process  Interdisciplinary approach throughout the process of care  Community-based rehabilitation  Longer therapy sessions  Holistic approach  Group therapy sessions  Availability of rehabilitation staff  Promote health facility in the community |
| Physical environment | Aspects of the physical environment, such as infrastructure, weather, and travelling conditions, may act as barriers or facilitators to accessing health services. | Transportation difficulties  Harsh weather conditions  Physical inaccessibility |  |
| Disability awareness | Factors associated with being educated and aware about disabilities and the etiology of health conditions | Lack of awareness | Disability awareness activities  Educating caregivers about child's condition  Advocacy in the community |
| Communication | Factors that affect access to health care due to communication with healthcare staff or the communication of information | Inaccessible resources  Communication barriers |  |
| Social factors | Aspects of the individual’s social environment, such as social support and relationships, that may affect access to health services | Lack of privacy  Lack of support  Parental neglect  Lack of time | Motivational support groups for caregivers  Peer support |
| Finance | Economic factors, such as poverty and funding, may affect a child’s ability to access health services | Poverty  Lack of funds | Incentives given to family at health facility |

**C: Barriers to Healthcare Services Access for CwDs**

| **Barrier/s** | **Definition** | **Papers discussing the barrier** |
| --- | --- | --- |
| Stigma | Stigma towards disability may deter parents from seeking treatment for their children. Caregivers often avoid being open about their child’s condition out of fear of being stigmatized. This includes experienced stigma as well as fear of being stigmatized. | (12, 13, 15, 23) |
| Cultural beliefs | Some cultures have different beliefs about disability and the etiology of health conditions, such as religious punishments or witchcraft | (13, 19, 21, 23, 25) |
| Negative attitudes | The personal attitudes of family members, health staff, or the community in which the individuals live may be negative towards disability or seeking treatment | (11, 17, 19, 20, 21) |
| Lack of health services | There is an absence of needed health services, including not enough clinics and hospitals. | (15, 20, 21) |
| Inadequate health care professionals | Health care staff are not adequately trained to deal with disability | (16, 17, 21, 23, 25) |
| Lack of qualified professionals | There are not enough health care professionals in a health facility; the facility is understaffed | (11, 17, 22, 23) |
| Lack of resources and equipment | The health care facility is lacking resources and equipment required to treat children with disabilities | (11, 16, 17, 22) |
| Bad reputation of health facility | The reputation of the health facility can deter people from accessing the facility. | (23) |
| Transportation difficulties | Travelling conditions make it difficult for the caregivers to travel with their children. This may be due to the nature of the child’s condition or the health care facility may be too far away from the family’s home. | (25, 16, 17, 22, 23) |
| Physical inaccessibility | Health services are difficult to access due to infrastructure (ex: no ramps for wheelchairs) | (20) |
| Harsh weather conditions | Weather (such as heavy rain or flooding) acts as a barrier by making it difficult to access health facilities | (23) |
| Lack of awareness | There is a lack of awareness regarding child disability. Disability is often unrecognized in children and the needs of children who are diagnosed often go unconsidered. This lack of awareness can come from the child’s family, community, or even the health care staff | (11-13) |
| Communication barriers | Health care staff is not able to communicate with children with disabilities (ex: staff doesn’t know sign language) | (20) |
| Inaccessible resources | Resources such as ads and print materials are not accessible by people with disabilities (ex: resources not available in braille) | (20) |
| Lack of privacy | Many people with disabilities require the help of a third person to access health services (ex: a translator for sign language) and this can prevent them from accessing services due to the lack of privacy | (20) |
| Lack of support | Lack of support or discouragement in general from peers, family, the community, etc. regarding accessing health care or seeking treatment | (21) |
| Parental neglect | Parents/caregivers are not taking their children to health services as a result of child abuse/neglect | (17) |
| Lack of time | Caregivers don't have time to take their children to health facilities (ex: being unable to take time off from work) | (15, 23) |
| Lack of funds | The health facility or treatment program is underfunded. This may be due to a lack of funds from the government allocated to healthcare services for the children | (11, 13, 16, 18, 22) |
| Poverty | The family of the child with a disability is experiencing financial difficulties that make it difficult to access health services | (15, 17, 20-23) |

**D: Facilitators to Healthcare Services Access for CwDs**

| **Facilitators** | **Definition** | **Papers** |
| --- | --- | --- |
| Disability awareness activities | Activities that promote awareness to a disability or health condition | (25) |
| Motivational support groups for caregivers | Support groups for families or caregivers of disabled children helps to inform and empower them. These support groups can be through a hospital, health care facility, or within the community | (17, 21) |
| Peer support | Psychosocial/emotional and social support from other caregivers, family members, healthcare staff, etc. | (13, 15, 23) |
| Caregivers and children are active participants in the care process | Caregivers and children are not just passive recipients of care but actually have a say in the process of care | (21) |
| Interdisciplinary approach throughout the process of care | Professionals from different disciplines collaborate to provide the best care possible | (21) |
| Hopefulness regarding treatment | Caregivers/family notice an improvement in the child's health and this gives them a sense of hope that encourages them to continue (or start) seeking treatment or to support the child | (15, 21) |
| Advocacy in the community | Parents advocate for their child in their community by letting the community see their children as not something to be scared of. This works to decrease stigma. | (21) |
| Community-based rehabilitation (CBR) | Community-based rehabilitation is rehabilitation treatment that takes place in the community or in the patient’s home as opposed to a hospital or health facility. | (14) |
| Incentives given to family at health facility | Incentives provided at the health care facility encourage parents to access that facility (Ex: toys, transportation money, aids, etc.) | (15) |
| Longer therapy sessions | Longer therapy sessions allow the caregivers to consult more with the healthcare provider | (15) |
| Holistic approach | Providing all rehabilitation services in one area (ex: in the same hospital) | (15) |
| Group therapy sessions | Therapy sessions are provided in groups and this allows caregivers to support each other and form better relationships with each other and with the rehab staff. | (15) |
| Availability of rehabilitation staff | The rehabilitation staff is able to provide the required services each time patients visit the health facility | (15) |
| Community workers and volunteers | Health workers and volunteers in the community work to identify children with disabilities | (23) |
| Promote health facility in the community | Members in the community share positive stories about the health facility and this may encourage others to use the facility | (23) |
| Educating caregivers about child's condition | Educating caregivers about the child's condition helps to foster care and acceptance and works to decrease child neglect and stigma | (17, 21) |
